# Monitoring seasonal and long-term forest canopy dynamics using Sentinel-2 imagery and Random Forest on the Google Earth Engine platform

**DOI:** 10.1371/journal.pone.0361024

**Published:** 2026-10-21

**Authors:** Pavan Kumar, Rahul Kumar, Soumyajeet Sahu, Manmohan Dobriyal, Pankaj Lavania, Manish Srivastav, Prem Chandra Pandey, Benson Turyasingura

**Affiliations:** 1 Department of Forest Resource Management, College of Horticulture and Forestry, Rani Lakshmi Bai Central Agricultural University, Jhansi, India; 2 Centre of Geospatial Science and Technology, College of Horticulture and Forestry, Rani Lakshmi Bai Central Agricultural University, Jhansi, India; 3 Centre of Geospatial Science and Technology, College of Horticulture and Forestry, Rani Lakshmi Bai Central Agricultural University, Jhansi, India; 4 Department of Silviculture and Agroforestry, College of Horticulture and Forestry, Rani Lakshmi Bai Central Agricultural University, Jhansi, India; 5 Department of Forest Resource Management, College of Horticulture and Forestry, Rani Lakshmi Bai Central Agricultural University, Jhansi, India; 6 College of Horticulture and Forestry, Rani Lakshmi Bai Central Agricultural University, Jhansi, India; 7 Department of Life Sciences, School of Natural Sciences (SoNS), Shiv Nadar Institution of Eminence (Deemed to be University), Greater Noida, India; 8 Faculty of Agriculture and Environmental Sciences, Department of Environment and Natural Resources, Kabale University, Kabale, Kampala, Uganda; Central University of Rajasthan, INDIA

## Abstract

Understanding forest canopy dynamics is critical for assessing the condition, resilience, and conservation priorities of protected landscapes. This study evaluated the spatiotemporal and seasonal variation in mapped forest canopy density within the Orchha Wildlife Sanctuary, Madhya Pradesh, India, using multi-seasonal Sentinel-2 imagery and a Google Earth Engine (GEE)-based Random Forest (RF) classification framework. Sentinel-2 data for pre-monsoon (March–May) and post-monsoon (October–December) periods in 2016 and 2024 were analyzed independently. Canopy-density information was derived from NDVI, NDMI, elevation, slope, and GLCM texture features and classified into four classes: non-forest, open, moderate, and dense canopy. The same RF procedure was applied to all four season-year datasets using NDVI-informed canopy-density strata and balanced reference sampling. Overall accuracy ranged from 97.80% to 98.95%, with Kappa coefficients of 0.970–0.986. The results indicated clear seasonal and interannual redistribution of mapped canopy-density classes. During the pre-monsoon period, non-forest and open classes decreased by 39.62% and 7.15%, respectively, while moderate and dense classes increased by 231.64% and 240.43%. During the post-monsoon period, moderate canopy increased by 26.01%, whereas dense canopy decreased by 9.46%. Within the common comparison area of 4,533.54 ha, transition analysis revealed substantial class persistence and redistribution, particularly among adjacent canopy-density classes such as open-to-moderate and dense-to-moderate transitions. Spatial changes were distributed across riparian, interior, and peripheral zones. Because 2016 used Level-1C top-of-atmosphere data and 2024 used Level-2A surface-reflectance products, interannual differences should be interpreted as changes in mapped canopy-density classes rather than precise biophysical changes in canopy structure. The framework provides a replicable approach for seasonal forest monitoring and spatially targeted conservation planning.

## 1. Introduction

Forests are among the most vital terrestrial ecosystems, serving as biodiversity reservoirs, carbon sinks, climate stabilizers, and regulators of hydrological and biogeochemical cycles [1–3]. As the sustainable management of forested and wetland ecosystems becomes more critical, integrated and high-resolution monitoring tools are needed to detect ecosystem degradation and to determine spatiotemporal changes in vegetation conditions and to support evidence-based approaches to conservation and management planning [4].They contribute to multiple ecosystem services such as air purification, erosion control, and nutrient cycling, making them crucial to both environmental health and human livelihoods [5,6]. In the context of global environmental change, forests especially in tropical and subtropical regions are under increasing pressure from deforestation, fragmentation, and climate variability [7,8]. Forest canopy density, a measure of the vertical and horizontal structure of tree cover, is a key ecological indicator that reflects the health, maturity, and resilience of forest landscapes [9]. Canopy cover influences photosynthetic activity, soil moisture retention, and understory microclimate, all of which are essential for forest regeneration and species composition (Raymond [10]. Hence, spatiotemporal canopy cover dynamics must be monitored to gain insight into ecological responses to climatic variability, anthropogenic pressures, and natural disturbances.Multi-temporal remote sensing techniques are useful to quantify vegetation structure and condition changes both spatially and temporally, allowing for detection of areas of canopy loss, canopy regeneration, and structural change.This long-term geospatial monitoring is especially critical in environments with varying heterogeneity and seasonal change, where the reaction of vegetation can also significantly differ in response to climate, moisture, and land-use pressure [11–13].Multi-temporal satellite imagery has thus been established as an important tool for quantitative assessment of landscape changes in order to identify landscape drivers, evaluate landscape responses and support evidence-based forest conservation and management for a variety of geographical regions [14].

In regions facing increasing land use change and climatic stress such as India’s tropical dry forests tracking canopy density changes can inform sustainable forest management and conservation strategies [15–17]. These dry deciduous ecosystems are particularly vulnerable due to their seasonal water availability, fire susceptibility, and proximity to expanding agricultural frontiers [18,19]. Moreover, habitat degradation in wildlife sanctuaries poses threats to biodiversity and also compromises ecological connectivity, the landscape connectivity analysis is therefore essential for sustainable conservation planning [20–22].The study of forest ecosystems has been transformed by remote sensing which allows consistent, repeatable and synoptic observations across time [23–25].With the rapid development of LiDAR technology [26,27], especially in adaptive energy control of lasers and optimization of echo signals, the accuracy and reliability of LiDAR remote sensing observations under complex environmental conditions have been greatly enhanced [28].In addition, through the effective suppression of internal stray light, the optical system of LiDAR has been improved, improving the quality of the data and the accuracy of measurement, which has laid the foundation for using it in various environmental monitoring applications with higher reliability [28–30].The recent advancements in point-cloud reconstruction have also boosted the understanding of vegetation-occluded terrain features, which has led to more accurate structural characterization of complex landscapes [31]. The integration of LiDAR with optical imagery and artificial intelligence [32] techniques has further improved feature extraction, object detection, and land-cover characterization in complex environmental conditions [17,26,27,33]. The Sentinel-2 satellite mission, developed by the European Space Agency under the Copernicus program, offers high spatial (10–60 m), temporal (5-day revisit), and spectral (13 bands) resolution imagery, making it highly suitable for vegetation monitoring and canopy analysis [34,35]. Sentinel-2’s red-edge, shortwave infrared (SWIR), and near-infrared (NIR) bands are particularly effective in distinguishing vegetation types and assessing forest structure, vigor, and moisture conditions across different seasons [36,37]. When combined with vegetation indices such as the Normalized Difference Vegetation Index (NDVI), Enhanced Vegetation Index (EVI), and Normalized Difference Moisture Index (NDMI), Sentinel-2 imagery allows for enhanced interpretation of forest canopy conditions [38–40]. In recent years, deep learning methods with Sentinel-1 SAR observations have also been shown to be highly accurate and automated tools for detecting environmental features, highlighting the growing significance of artificial intelligence in contemporary remote sensing applications [41–43].To enhance image correction, feature enhancement, and improve accuracy of image interpretation, artificial intelligence techniques are being increasingly utilized for many earth observation datasets [44].

The latest developments in deep convolutional neural networks (DCNN), including encoder–decoder models like ConvNeXt-UNet, have significantly improved the extraction of features and segmentation of images from Earth observation datasets, highlighting the increasing role of AI in remote sensing applications [45,46].Unmanned aerial vehicle (UAV)-based remote sensing is also an enabling technology for high-resolution spatial information collection and enhancing the accuracy of environmental monitoring, in addition to satellite-based remote sensing [47].

Complementing remote sensing data with machine learning [48] classifiers particularly Random Forest (RF) further improves the accuracy and scalability of land cover classification [42,49–51].With the recent advances of large models of multimodal artificial intelligence, automated image segmentation and feature extraction have also seen significant improvements in the field of agricultural and environmental remote sensing [42].RF is an ensemble learning method capable of handling nonlinear relationships and multi-source predictor variables while minimizing overfitting [16]. Its integration within cloud computing platforms such as Google Earth Engine (GEE) facilitates large-scale processing, automated workflows, and near-real-time spatiotemporal comparisons [51,52]. Machine learning approaches in GEE allow for the fusion of spectral, temporal, and ancillary datasets such as topography, soil type, and texture metrics thereby increasing classification robustness [53,54]. Several studies have demonstrated the effectiveness of this combined approach in delineating forest types, detecting canopy gaps, and mapping disturbance events across heterogeneous landscapes [55].

This study applies multi-temporal Sentinel-2 imagery and Random Forest classification to assess forest canopy density changes in Orchha Wildlife Sanctuary, a protected area in the Tikamgarh district of Madhya Pradesh, India. The sanctuary spans approximately 46 km^2^ and is characterized by mixed deciduous forests, scrublands, seasonal wetlands [4], and riverine ecosystems along the Betwa River [56]. With its subtropical semi-arid climate and clear seasonal demarcation summer (March–June), monsoon (July–September), and winter (October–February) Orchha presents an ideal case for examining seasonal and decadal forest dynamics [57]. The analysis focuses on the years 2016 and 2024, using both pre-monsoon and post-monsoon datasets to capture phenological variations and structural shifts in canopy cover. By integrating vegetation indices (NDVI, NDMI, EVI), topographic features (elevation, slope, aspect), and texture metrics (GLCM-based indicators) into a Random Forest framework on GEE, this study provides a robust, high-resolution assessment of canopy change across time and space [58,59]. This methodological synergy ensures enhanced classification accuracy and ecological interpretation, particularly in heterogeneous and fragmented forested landscapes. The study aims to analyze seasonal and decadal changes in forest canopy density in Orchha Wildlife Sanctuary from 2016 to 2024 using Sentinel-2 data and Random Forest classification. It focuses on identifying spatial patterns of canopy gain and loss to support sustainable forest management.

## 2. Materials and methods

### 2.1 Study area

The Orchha Wildlife Sanctuary is situated in the Tikamgarh district of Madhya Pradesh, India, along the banks of the Betwa River, a major tributary of the Yamuna. The sanctuary was established in 1994 and is administered by the Madhya Pradesh Forest Department. It lies within the Bundelkhand region, a semi-arid plateau that represents a transitional zone between the Gangetic Plains and the Vindhyan highlands (Fig 1). The sanctuary comprises a heterogeneous landscape featuring a mosaic of tropical dry deciduous forests, open scrublands, riverine corridors, riparian vegetation, grasslands, and seasonal wetlands. This ecological diversity supports a wide array of flora and fauna, including several species of conservation concern, such as the Indian gazelle (Gazella bennettii), sloth bear (Melursus ursinus), and a variety of resident and migratory bird species. The region is characterized by a subtropical climate with three well-defined seasons: a hot and dry summer from March to June, a monsoon season from July to September, and a cool winter extending from October to February. The average annual precipitation is approximately 1000 mm, most of which is received during the monsoon months. These distinct seasonal phases lead to substantial intra-annual variability in vegetation structure, phenology, and moisture availability factors that directly influence forest canopy density and composition. The sanctuary's seasonally dynamic environment and vulnerability to anthropogenic pressures, including encroachment, grazing, and mining activities in the surrounding buffer zones, make it a critical area for ecological monitoring. Its diverse habitat types and pronounced seasonal changes provide an ideal natural laboratory for investigating spatiotemporal variations in forest canopy dynamics, particularly in relation to climatic variability and human-induced disturbances.

**Fig 1:**
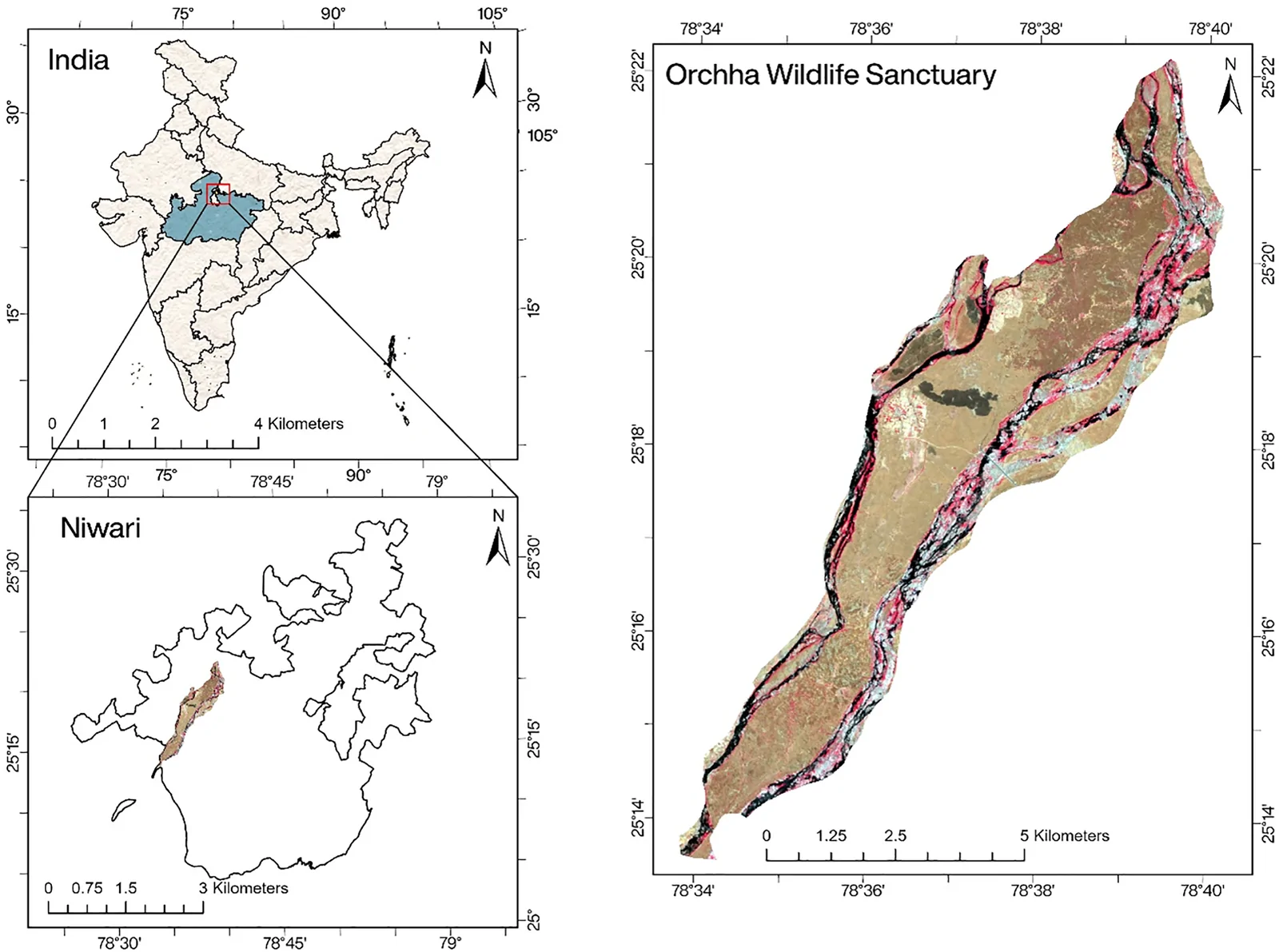
Study area map of Orchha Wildlife Sanctuary(Author generated image and map, No copy right submission required).

The vegetation of the sanctuary corresponds to the Dry Teak Forest (5A/C1b) and the Anogeissus pendula (Kardhai) Forest (5/E1) types of the Champion and Seth [19] classification. Teak (*Tectona grandis*) and kardhai (*Anogeissus pendula*) form the dominant canopy, with kardhai prevailing in the northern part of the sanctuary and teak in the southern part, while *Adina cordifolia*, *Lagerstroemia parviflora*, *Terminalia tomentosa*, *Acacia catechu*, *Buchananialanzan* and *Madhuca indica* occur scattered throughout the area. Riverbanks and stream beds along the Betwa and Jamni rivers are occupied mainly by *Syzygiumcumini* and *Terminalia arjuna*. A floristic survey of the sanctuary recorded 42 angiosperm genera belonging to 23 families, with Leguminosae the most represented family [60]. The dominant species, their associated forest types and their principal ecological characteristics are summarised in Table 1. These compositional differences are directly relevant to the interpretation of canopy density dynamics, because the deciduous phenology and canopy architecture of the constituent species govern the magnitude of the seasonal NDVI signal recorded by Sentinel-2.

**Table 1:** Dominant tree species and associated vegetation communities of Orchha Wildlife Sanctuary.

Dominant species	Forest type [19]	Ecological characteristics relevant to canopy dynamics
*Tectona grandis* (teak)	Dry Teak Forest (5A/C1b)	Large-leaved dominant of the southern blocks; strongly deciduous, sheds leaves during the dry season and flushes rapidly after the monsoon, producing a large seasonal amplitude in canopy greenness
*Anogeissus pendula* (kardhai)	Anogeissus pendula Forest (5/E1)	Dominant on shallow, rocky Vindhyan soils in the northern blocks; small-leaved and drought-hardy, forming low to moderately dense closed canopy with a smaller seasonal greenness amplitude
*Adina cordifolia*, *Lagerstroemia parviflora*, *Terminalia tomentosa*	Associates within 5A/C1b and 5/E1	Scattered upper- and mid-storey associates that contribute to canopy heterogeneity and to a mixed spectral response within the moderate and high density classes
*Acacia catechu*, *Buchananialanzan*, *Madhuca indica*	Associates within 5A/C1b and 5/E1	Drought-tolerant associates of open and degraded stands; typically fall within the low and moderate canopy density classes
*Syzygiumcumini*, *Terminalia arjuna*	Riparian corridor of the Betwa and Jamni rivers	Evergreen to semi-evergreen riparian species with sustained access to soil moisture; maintain high canopy density and greenness through the dry season

### 2.2 Satellite datasets

This study utilized Sentinel-2 optical imagery acquired from EarthExplorer archive of the United States Geological Survey (USGS) (https://earthexplorer.usgs.gov/) during both the pre-monsoon (March-May) and post-monsoon (October–December) seasons for the years 2016 and 2024, enabling both seasonal and decadal-scale comparisons of forest canopy dynamics. These data are available to the public via the USGS EarthExplorer (https://earthexplorer.usgs.gov/). The Sentinel-2 constellation, comprising Sentinel-2A and Sentinel-2B satellites, offers high-resolution multispectral imagery across 13 spectral bands with spatial resolutions ranging from 10 to 60 meters and a revisit frequency of approximately five days, making it well-suited for vegetation monitoring and change detection (Table 2).

**Table 2:** Satellite data and sensors used in study.

Year	Season	Platform	Product	Resolution	Use
2016	Pre-Monsoon	Sentinel-2A	Level-1C	10–60 m	NDVI, classification input
2016	Post-Monsoon	Sentinel-2A	L1C / TOA	10–60 m	NDVI, NDMI, contrast metrics
2024	Pre-Monsoon	Sentinel-2A/B	Surface Reflectance (SR)	10–60 m	NDVI, NDMI, texture, RF model
2024	Post-Monsoon	Sentinel-2A/B	SR (L2A)	10–60 m	RF classification change map

For 2024, Level-2A Surface Reflectance products were used, whereas the 2016 analysis used Level-1C Top-of-Atmosphere reflectance because surface-reflectance products were not available for the early Sentinel-2 acquisition period used in this study. This difference in processing level introduces a potential source of interannual radiometric inconsistency. Cloud and cirrus contamination in the 2016 imagery was reduced using the QA60 cloud mask, while the 2024 imagery was screened using the scene classification layer. Consequently, the resulting 2016–2024 differences were interpreted as changes in mapped canopy-density classes rather than as direct quantitative estimates of biophysical canopy change.

### 2.3 Ancillary data

In addition to the core Sentinel-2 multispectral imagery, several ancillary datasets were integrated to enhance classification accuracy and to capture the structural and ecological characteristics of the forest canopy. Topographic variables, including elevation and slope, were derived from the Shuttle Radar Topography Mission (SRTM) Digital Elevation Model (DEM) at a spatial resolution of 30 meters. These variables play a crucial role in explaining vegetation distribution patterns and microclimatic variation, especially in heterogeneous landscapes like Orchha Wildlife Sanctuary, where elevation gradients influence species composition and canopy density. To incorporate textural heterogeneity and improve discrimination of forest classes, texture features were extracted using the Gray-Level Co-occurrence Matrix (GLCM) method [53]. Specifically, GLCM contrast was computed from the Near-Infrared (NIR) band (Band 8), which is sensitive to vegetation structure and biomass. A 3 × 3 moving window was applied to derive localized measures of spectral variability, which serve as proxies for forest complexity and canopy roughness. These texture metrics are particularly useful in separating dense forest from degraded or fragmented areas that may have similar spectral but differing structural signatures [28]. Two widely used vegetation indices were also calculated to capture canopy biophysical properties: The Normalized Difference Vegetation Index (NDVI) and the Normalized Difference Moisture Index (NDMI). NDVI, calculated as (B8 − B4)/ (B8 + B4), provides an estimate of photosynthetic activity and canopy greenness, while NDMI, derived from (B8 − B11)/(B8 + B11), reflects canopy moisture content and is useful in detecting water stress or senescence. Both indices were computed for each seasonal composite to capture intra-annual variation in vegetation dynamics. All ancillary layers, including elevation, slope, GLCM contrast, NDVI, and NDMI, were resampled or harmonized to match the spatial resolution of the Sentinel-2 imagery. These layers were incorporated as explanatory variables in the Random Forest classification models for both 2016 and 2024 across pre-monsoon and post-monsoon periods. The combined use of spectral, structural, and topographic information aimed to improve the discrimination of forest canopy conditions and enhance model robustness across different seasons and time periods [37].

### 2.4 Image preprocessing

For both the 2016 and 2024 study periods, cloud-free Sentinel-2 imagery was acquired using the Google Earth Engine (GEE) platform, targeting the pre-monsoon (March-May) and post-monsoon (October–December) seasons. These specific seasonal windows were selected to capture vegetation conditions before and after the monsoon rainfall, which significantly influences canopy structure and greenness in tropical deciduous ecosystems. To ensure temporal consistency, a multi-date image collection was filtered by season and year. All scenes with less than 10% cloud cover over the study area were retained for further processing. For both study years, Sentinel-2 imagery was processed in Google Earth Engine (GEE) for the pre-monsoon (March–May) and post-monsoon (October–December) periods. The image collections were filtered spatially to the Orchha Wildlife Sanctuary and temporally to the respective seasonal windows. For 2016, scenes with less than 20% reported cloud cover were retained and cloud and cirrus contamination was reduced using the QA60 band. Because the 2016 archive contains scenes with differing band availability, only scenes containing the QA60 band were retained for the final compositing. For 2024, Level-2A Surface Reflectance imagery with less than 10% reported cloud cover was used, and cloud, cirrus, snow/ice, and cloud-shadow contaminated pixels were masked using the scene classification layer. All retained images were scaled to reflectance before calculation of the vegetation indices and texture metrics. (Fig 2).

**Fig 2:**
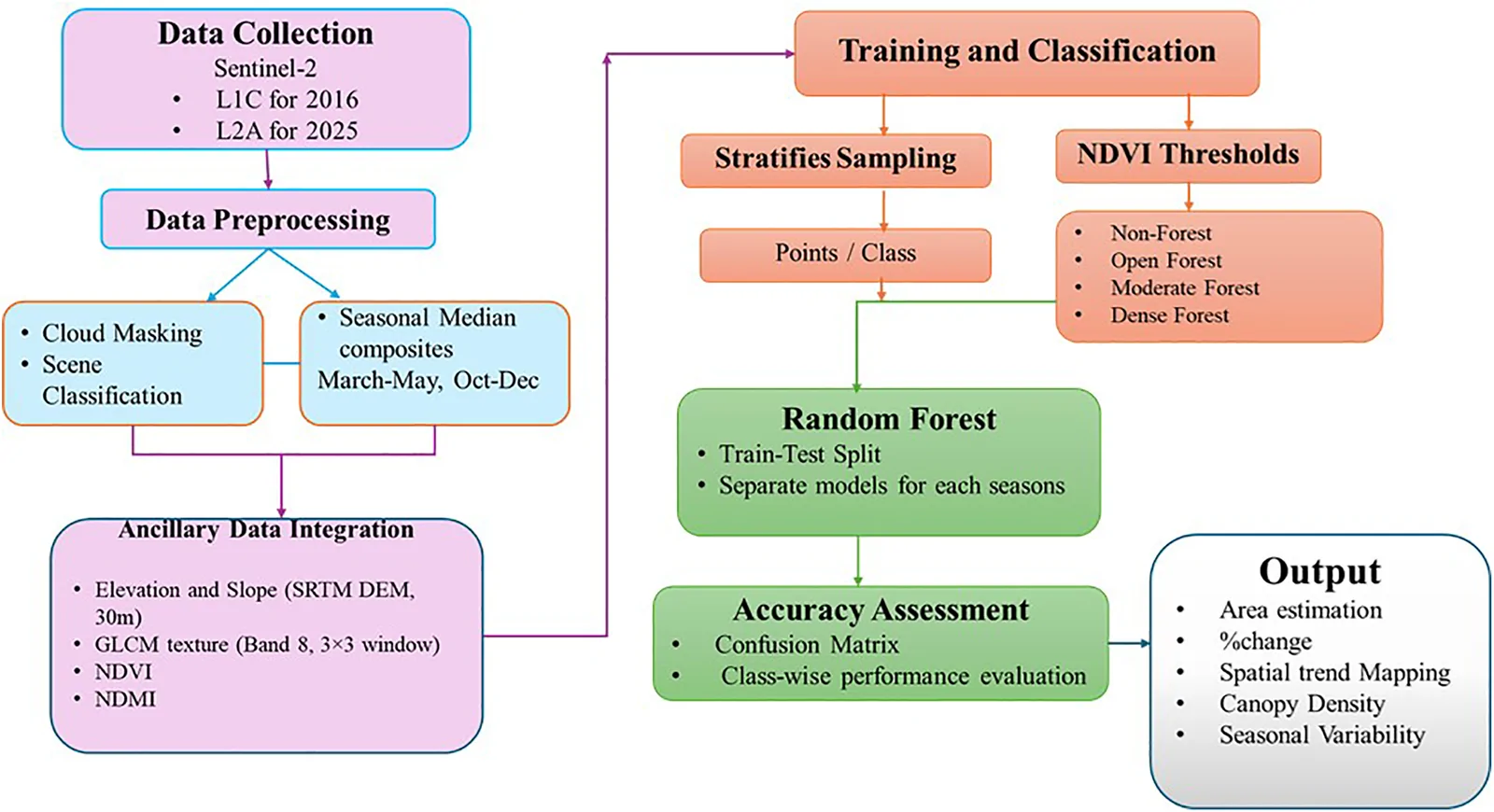
Methodology flowchart adopted in the study.

To generate representative seasonal composites, all qualifying images for each season were processed using the median compositing technique, which reduces residual cloud effects and temporal noise by calculating the median reflectance value for each pixel across the image stack. These composites were subsequently clipped to the administrative boundary of Orchha Wildlife Sanctuary to ensure spatial alignment and eliminate extraneous areas. The resulting seasonal mosaics served as the primary input for vegetation index calculation and classification analysis in the subsequent workflow.

### 2.5 GLCM-based texture metrics in forest canopy analysis

Texture is a critical spatial characteristic in remote sensing that reflects the arrangement and relationship of pixel intensities in an image. In forest monitoring, texture features can capture structural patterns such as canopy roughness, heterogeneity, and density gradients, which are often not fully represented by spectral information alone [61]. The Gray-Level Co-occurrence Matrix (GLCM) is a widely used statistical method for deriving texture metrics from imagery, particularly effective in analyzing vegetated landscapes. GLCM computes how often pairs of pixel values (Gray levels) occur at a specified spatial relationship (e.g., horizontal, vertical, diagonal) within a moving window across an image [54]. From this matrix, various second-order statistical texture features can be extracted, including contrast, correlation, energy, and homogeneity. Among these contrasts is particularly valuable for vegetation studies, as it quantifies the local variation in pixel values and is sensitive to spatial heterogeneity in canopy structure. In this study, GLCM-based contrast values were derived from the Near-Infrared (NIR) band (Band 8) of Sentinel-2 imagery using a 3 × 3 moving window. The NIR band was selected due to its strong reflectance from healthy vegetation and sensitivity to leaf and canopy structure. The resulting texture layer was used as an ancillary predictor variable in the Random Forest classification to enhance the discrimination between forest canopy density classes. Incorporating GLCM contrast allowed the model to capture subtle spatial differences that are not apparent through spectral indices alone, such as the fine-scale variability between low, moderate, and high canopy density zones. For instance, densely forested areas tend to show smoother texture patterns (low contrast), whereas open or degraded forests exhibit higher spatial variability (high contrast), improving class separability. The combined spectral, textural, and topographic predictors were used to provide complementary information for discriminating canopy-density classes across seasons and years.

### 2.6 Forest Canopy Density (FCD) classification

A stratified sampling strategy was used to obtain balanced reference data for the four canopy-density classes. The classes were defined from the NDVI thresholds: non-forest (NDVI ≤ 0.10), open forest (0.10 < NDVI ≤ 0.25), moderate canopy (0.25 < NDVI ≤ 0.38), and dense forest (NDVI > 0.38). Seventy-five reference samples were obtained for each class, giving a total of 300 samples for each season-year dataset. These samples were used to train and validate an independent Random Forest model for each combination of year and season. The same classification procedure was therefore applied to 2016 pre-monsoon, 2016 post-monsoon, 2024 pre-monsoon, and 2024 post-monsoon datasets. Each RF model was configured with 50 decision trees, selected based on empirical tests for computational efficiency and performance stability (Fig 3). A 70:30 train–test split was employed, where 70% of the data were used to train the classifier, and the remaining 30% was reserved for validation. Model performance was quantitatively assessed using confusion matrices, from which standard classification accuracy metrics were derived, including overall accuracy, user accuracy, producer accuracy, and the Kappa coefficient. These metrics provided insights into the reliability of the classification across all canopy classes and helped identify potential sources of misclassification. This stratified and seasonally disaggregated approach enabled a nuanced and temporally sensitive assessment of canopy density changes within the Orchha Wildlife Sanctuary.Because the canopy-density strata were defined using NDVI thresholds and NDVI was also included as a predictor variable in the Random Forest models, the resulting accuracy statistics represent agreement between the RF predictions and the NDVI-informed reference strata rather than fully independent validation of field-measured canopy density. The accuracy values are therefore interpreted as an assessment of classification consistency within the defined canopy-density scheme rather than as direct validation of biophysical canopy structure.

**Fig 3:**
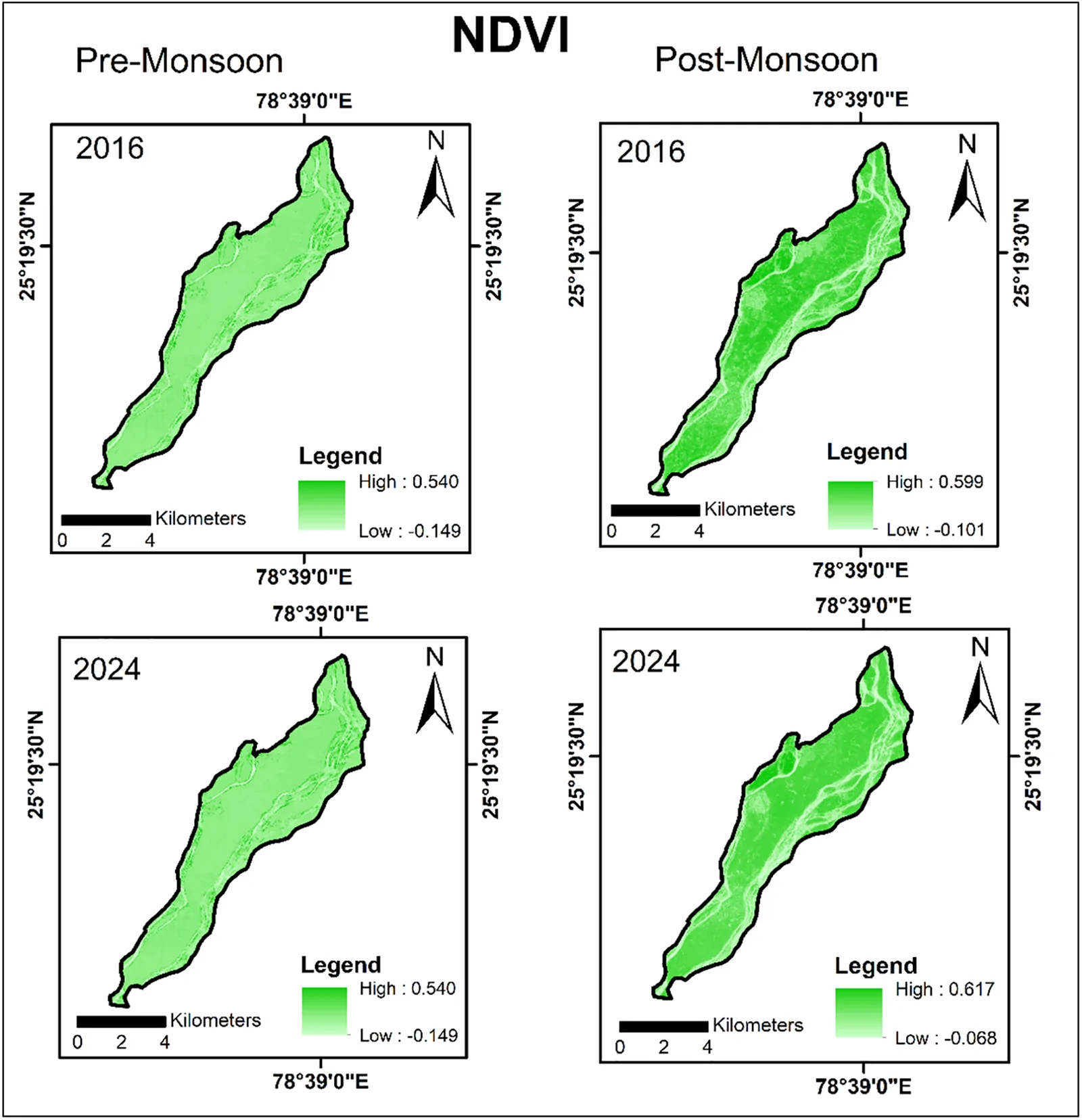
NDVI map of different seasons for the study site(Author generated image and map, No copy right submission required).

The rationale for the selected NDVI thresholds is as follows. The class boundaries were not transferred unchanged from any single published study. They were adapted from NDVI ranges reported for canopy cover and forest density assessment in semi-arid and tropical dry deciduous stands [9,18,55] and were then adjusted to the NDVI distributions of the Sentinel-2 seasonal composites of the sanctuary through exploratory inspection of those composites together with visual interpretation of high-resolution imagery, so that each stratum corresponded to a visually distinguishable level of crown closure. The four resulting strata were also checked for correspondence with the canopy density classes used for national forest cover reporting in India, in which open forest, moderately dense forest and very dense forest are separated at crown densities of approximately 10, 40 and 70 per cent [56,57]. No independent field measurement of crown density was available for threshold calibration, so the thresholds are empirical rather than field-calibrated. Because they were tuned to the composites of this sanctuary, they are specific to this site, to Sentinel-2 and to the seasonal windows analysed here, and they should be re-derived rather than transferred directly to other areas, sensors or seasons. The limitations that follow from an NDVI-based stratification are discussed in Section 4.1.

The reference dataset comprised 300 points in total, with 75 points collected for each of the four canopy density classes, so that the design was balanced by class and no class dominated model training. The points were allocated by stratified random sampling within each canopy stratum across the full extent of the sanctuary rather than being clustered in a small number of blocks, and each point was labelled by visual interpretation of high-resolution imagery in conjunction with the NDVI value of the corresponding seasonal composite. The pooled set of 300 points was then partitioned randomly in a 70:30 ratio, giving approximately 210 points for model training and approximately 90 points for validation, that is, of the order of 53 training and 22 validation points per class. The validation subset was withheld from model fitting entirely and was therefore independent of the training subset; the accuracy metrics reported in Section 3.1 were computed exclusively from these withheld points. The same sampling design and the same 70:30 partition were applied independently to each of the four season-year models, so that every model was validated against points that had not contributed to its own training. The composition of the training and validation sets is summarised in Table 3.

**Table 3:** Distribution of training and validation reference points by canopy density class.

Canopy density class	NDVI range	Reference points collected	Training points (70%)	Validation points (30%)
Non-forest	NDVI ≤ 0.10	75	53	22
Low canopy density (open forest)	0.10 < NDVI ≤ 0.25	75	53	22
Moderate canopy density	0.25 < NDVI ≤ 0.38	75	53	22
High canopy density (dense forest)	NDVI > 0.38	75	53	22
Total		300	210	90

Note: the per-class training and validation counts are the nominal values of a random 70:30 partition of the pooled reference set. Exact per-class counts vary by one or two points between the four season-year models because the partition is drawn independently for each model.

### 2.7 Accuracy assessment and area estimation

Accuracy assessment was performed using stratified random sampling to ensure balanced validation across canopy density classes. Independent validation samples were used to generate confusion matrices and to calculate standard accuracy metrics, including overall accuracy (OA), producer's accuracy, user's accuracy, and the Kappa coefficient. Overall accuracy was computed as follows:


$$\begin{array}{c} \text{OA}=\frac{\sum_{\text{i}=\text{1}}^{\text{k}}\text{nii}}{\text{N}} \end{array}$$

where ${\text{n}}_{\text{ii}}$ is the number of correctly classified samples in class (i),(k)is the total number of classes, and N is the total number of validation samples.The Kappa coefficient (k)was calculated to account for agreement occurring by chance, using:


$$\begin{array}{c} \text{k}=\frac{{\text{p}}_{\text{o}}-{\text{p}}_{\text{e}}}{\text{1}-{\text{p}}_{\text{e}}} \end{array}$$

where ${\text{p}}_{\text{o}}$is the observed agreement and ${\text{p}}_{\text{e}}$ is the expected agreement by chance. Values of (k)closer to 1 indicate stronger agreement between classified and reference data. Class-wise producer’s and user’s accuracy values were derived from confusion matrices and are reported in supplementary tables or the appendix.

For area estimation, classified pixel counts were converted to area in hectares. The area of each class ccc was calculated as:


$$\begin{array}{c} {\text{A}}_{\text{c}}={\text{N}}_{\text{c }}\times {\text{r}}^{\text{2}}\times \text{0}.\text{0001} \end{array}$$

where ${\text{N}}_{\text{c}}$ is the number of pixels in class c and r is the pixel resolution in meters. For Sentinel-2 data at 10 m resolution, this simplifies to:


$$\begin{array}{c} {\text{A}}_{\text{c}}={\text{N}}_{\text{c}}\times \text{0}.\text{01} \end{array}$$

yielding area in hectares by multiplying the pixel count by 0.01.

Change detection between 2016 and 2024 was assessed by computing absolute and percentage changes in area for each forest canopy density class. Absolute change was calculated as:


$$\begin{array}{c} \Delta \text{A}={\text{A}}_{\text{2024}}-{\text{A}}_{\text{2016}} \end{array}$$

and percentage change was computed using:


$$\begin{array}{c} \%\text{Change}=\frac{{\text{A}}_{\text{2024}}-{\text{A}}_{\text{2016}}}{{\text{A}}_{\text{2016}}} \end{array}$$

To examine spatial redistribution among canopy-density classes, a four-by-four transition matrix was generated separately for the pre-monsoon and post-monsoon periods. Each matrix records the area transitioning from each 2016 canopy-density class to each corresponding 2024 class. Transition analysis was restricted to pixels with valid classifications in both years, resulting in a common comparison area of 4,533.54 ha for each season. The transition matrices were expressed in hectares using the 10 m pixel area.

## 3. Results

### 3.1 Classification accuracy

The Random Forest (RF) models showed high internal agreement with the NDVI-informed reference strata across all four season-year datasets (Fig 4). The 2016 pre-monsoon model achieved an overall accuracy (OA) of 98.86% with a Kappa coefficient (κ) of 0.985, while the 2016 post-monsoon model achieved an OA of 98.70% and κ = 0.983. For 2024, the pre-monsoon model achieved an OA of 97.80% and κ = 0.970, whereas the post-monsoon model achieved an OA of 98.95% and κ = 0.986. Thus, OA ranged from 97.80% to 98.95%, while the Kappa coefficient ranged from 0.970 to 0.986 across the four datasets (Fig 4).

**Fig 4:**
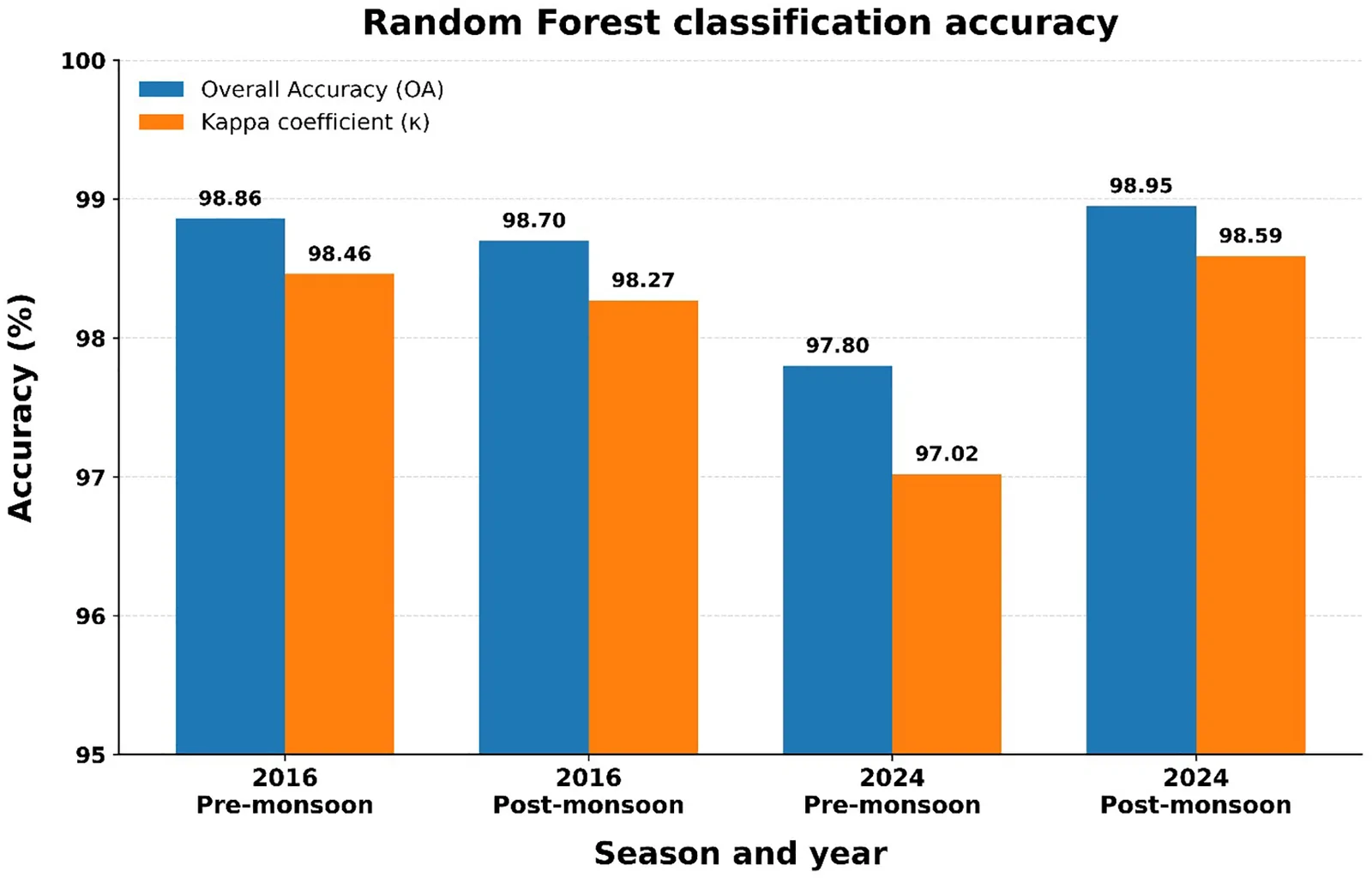
Classification accuracy graph comparing overall accuracy and kappa coefficient.

Class-wise producer's accuracy (PA) and user's accuracy (UA) were also generally high. For the 2016 pre-monsoon classification, PA was 100% for non-forest, open, and dense classes, and 96.55% for moderate canopy, while UA ranged from 95.65% to 100%. The 2016 post-monsoon classification similarly produced PA values of 95.24–100% and UA values of 94.74–100%, with the main discrepancy occurring in the open and moderate classes. For 2024, the pre-monsoon model showed a PA of 90.48% for the open class and a UA of 93.33% for the moderate class, while the remaining classes showed 100% PA or UA. The 2024 post-monsoon model achieved PA values of 95.45–100% and UA values of 96.30–100%. These results indicate that most classification errors involved confusion between adjacent canopy-density classes rather than substantial confusion with the non-forest class.

The relatively small discrepancies among the open and moderate canopy classes are consistent with their intermediate spectral characteristics, where gradual differences in vegetation density can produce overlapping spectral responses. Such overlap may be further influenced by seasonal vegetation conditions during the pre- and post-monsoon periods. However, the observed accuracy should be interpreted within the framework of the adopted reference-sampling procedure. Because the reference strata were defined partly using NDVI and NDVI was also included as a predictor in the RF models, the reported OA and Kappa values primarily indicate consistency with the NDVI-informed classification scheme rather than independent field-based validation of canopy structure.

Overall, the RF models produced consistent classification results across both years and seasons, providing a stable basis for mapping the four canopy-density classes. Nevertheless, the high internal accuracy should not be interpreted as evidence of equivalent accuracy in independent biophysical canopy measurements. Independent field observations or externally derived canopy-cover datasets would be required to assess the ability of the classification to represent actual structural canopy conditions.

### 3.2 Pre-monsoon forest canopy dynamics

The spatial distribution of mapped canopy-density classes during the pre-monsoon season showed substantial differences between 2016 and 2024 across Orchha Wildlife Sanctuary (Fig 5). The pre-monsoon period represents a relatively dry phase of the seasonal cycle, during which vegetation greenness and moisture availability can vary considerably in seasonally dry deciduous forests. In 2016, non-forest and open-canopy classes occupied much of the sanctuary, particularly along peripheral and fragmented areas, while moderate-canopy patches were distributed mainly across the central portion. Dense-canopy areas were comparatively limited and occurred primarily within the northeastern and northern forest patches. In 2024, the spatial distribution showed a marked redistribution toward moderate- and dense-canopy classes. The area classified as non-forest decreased from 1,241.92 ha in 2016 to 749.92 ha in 2024, representing a reduction of 492.00 ha (39.62%), while open canopy decreased from 2,988.16 to 2,774.40 ha (7.15%). In contrast, moderate canopy increased from 271.46 to 900.27 ha (231.64%), and dense canopy increased from 32.01 to 108.96 ha (240.43%). Thus, the pre-monsoon comparison indicates a pronounced redistribution of mapped area from non-forest and open-canopy classes toward moderate- and dense-canopy classes. This pattern may reflect differences in vegetation phenology and moisture conditions between the two observation periods; however, the interannual comparison should be interpreted cautiously because the 2016 analysis used Sentinel-2 Level-1C top-of-atmosphere data, whereas the 2024 analysis used Level-2A surface-reflectance data.

**Fig 5:**
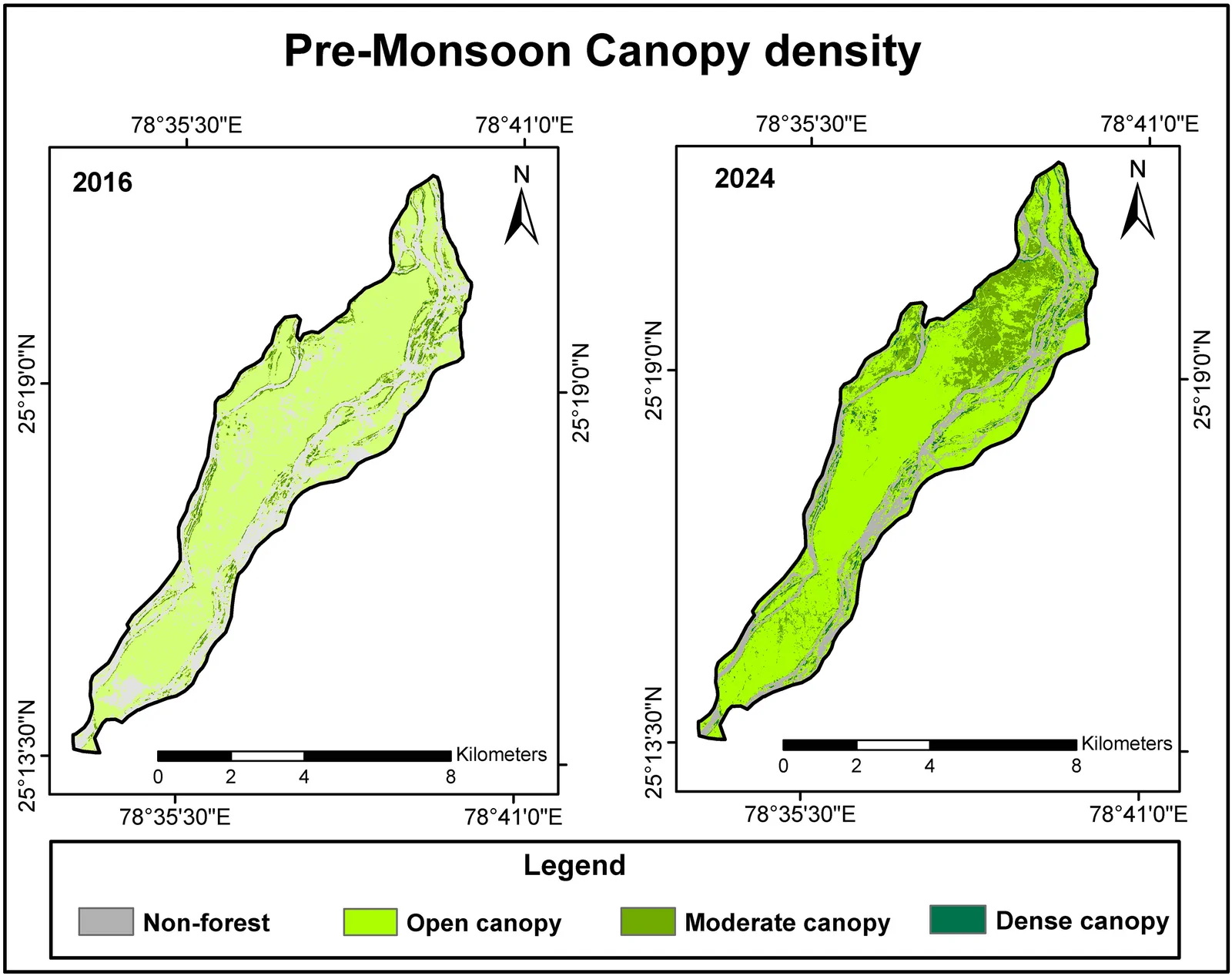
Pre-monsoon canopy-density distribution in Orchha Wildlife Sanctuary for 2016 and 2024, showing the spatial distribution of non-forest, open, moderate, and dense mapped canopy-density classes.

### 3.3 Climatic context and pre-monsoon 2016 canopy suppression

The pre-monsoon period (March–May) represents part of the hot and dry season in Orchha Wildlife Sanctuary, when seasonal water availability and vegetation phenology can strongly influence canopy greenness. The relatively low mapped canopy density observed in 2016 may therefore be consistent with antecedent moisture stress and seasonal phenological effects. However, rainfall and drought conditions were not quantitatively evaluated in this study; consequently, the observed 2016 pattern should be interpreted as consistent with potential climatic and moisture-related stress rather than directly attributed to a specific drought event.

The ecological composition of the sanctuary also provides an important context for interpreting the pre-monsoon spectral response. Tectona grandis (teak), which is prominent in the southern forest blocks, is strongly deciduous and sheds its leaves during the dry season, producing a large seasonal reduction in canopy greenness. In contrast, *Anogeissus pendula (kardhai)*, dominant in the northern blocks, is more drought-hardy and exhibits a comparatively smaller seasonal change in greenness. Other associated species, including Adina cordifolia, Lagerstroemia parviflora, and Terminalia tomentosa, contribute to heterogeneous canopy structure and mixed spectral responses, while drought-tolerant associates such as *Acacia catechu, Buchananialanzan, and Madhuca indica* commonly occur within open and moderate canopy conditions. These species-specific phenological differences may contribute to the spatial heterogeneity observed in the pre-monsoon canopy-density classification.

As shown in Fig 5, the 2016 pre-monsoon classification contained extensive non-forest and open-canopy areas, particularly toward peripheral and fragmented portions of the sanctuary, while moderate and dense canopy classes were more spatially restricted. The 2024 classification showed a redistribution toward moderate and dense mapped canopy classes. This difference may reflect changes in seasonal vegetation condition and antecedent moisture availability, but it may also be influenced by the different Sentinel-2 processing levels used for the two years. The persistence of lower-density areas in peripheral portions further indicates that canopy-density patterns remained spatially heterogeneous.The Betwa and Jamni river corridors provide an additional ecological contrast because *Syzygiumcumini and Terminalia arjuna* occur predominantly in these riparian environments and can maintain relatively high canopy greenness during the dry season through sustained access to soil moisture. Consequently, higher mapped canopy density in riparian areas during the pre-monsoon period may partly reflect differences in species composition and moisture availability rather than a uniform canopy response across the sanctuary. Overall, the 2016–2024 pre-monsoon comparison should therefore be interpreted as a combination of seasonal phenological variability, spatially heterogeneous vegetation composition, and interannual differences in mapped canopy-density classes, rather than direct evidence of climate-driven canopy suppression or subsequent forest recovery.

### 3.4 Post-monsoon forest canopy dynamics

The post-monsoon season represents a biologically active phase for the deciduous forest ecosystem of Orchha Wildlife Sanctuary, following the seasonal rainfall period. As shown in Fig 6, the comparison of mapped canopy-density classes between 2016 and 2024 indicates substantial spatial persistence together with localized redistribution among canopy-density classes. In 2016, a large proportion of the sanctuary was classified as moderate and dense canopy, with extensive higher-density patches particularly evident across the central and northern parts of the sanctuary. Lower-density and non-forest areas were more apparent along peripheral sections and fragmented portions of the landscape.In 2024, the overall spatial configuration remained broadly similar, although localized changes in canopy-density classes were evident. Quantitatively, the non-forest class remained nearly unchanged, increasing from 676.25 ha in 2016 to 680.77 ha in 2024 (0.67%). The area classified as open canopy decreased from 858.05 to 785.38 ha (8.47%), whereas moderate canopy increased from 991.73 to 1,249.70 ha (26.01%). In contrast, the dense-canopy class decreased from 2,007.52 to 1,817.69 ha (9.46%). Thus, the post-monsoon results indicate a redistribution toward the moderate-canopy class rather than a uniform expansion of the highest-density canopy class.

**Fig 6:**
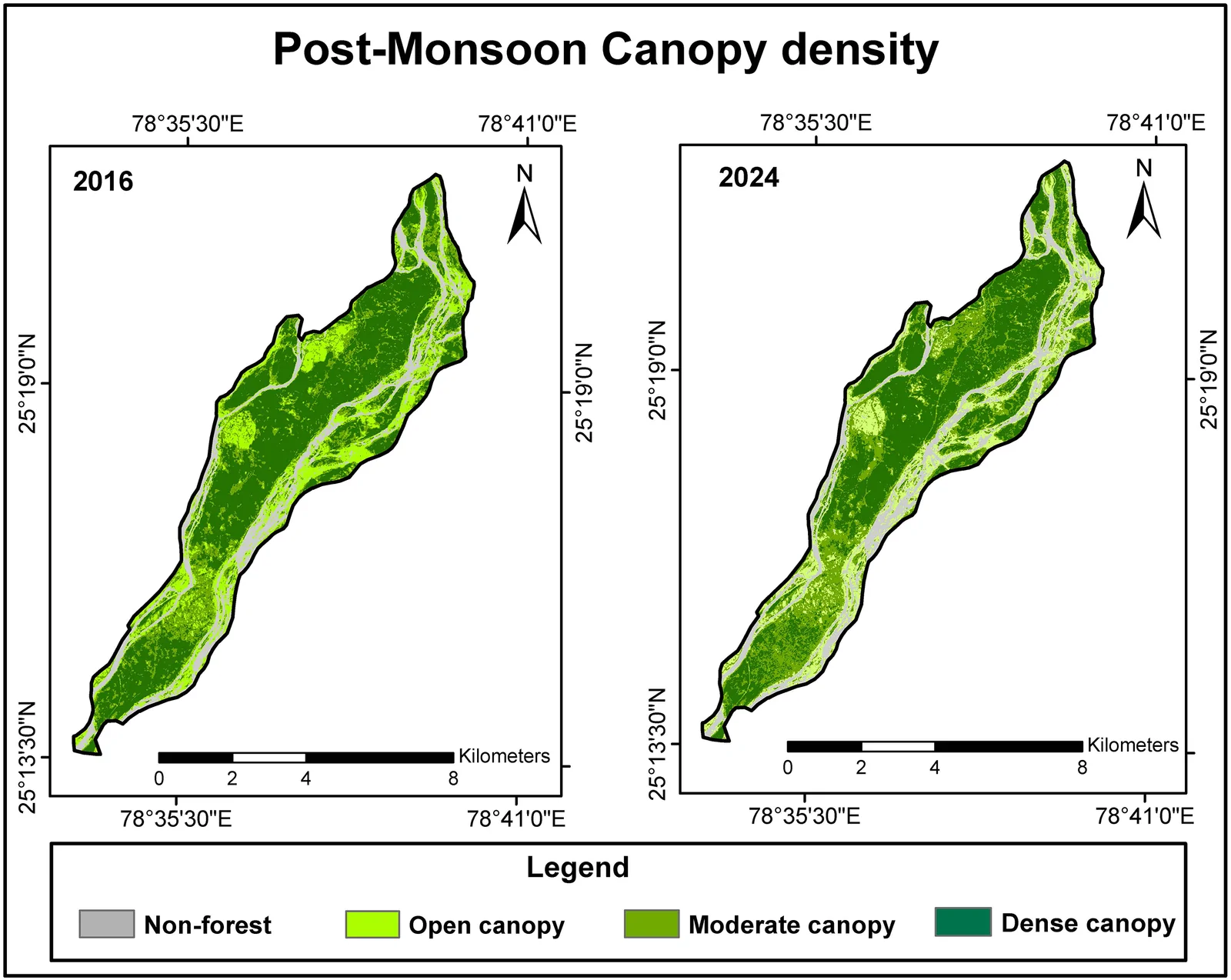
Post-monsoon canopy-density distribution in Orchha Wildlife Sanctuary for 2016 and 2024, showing the spatial distribution of non-forest, open, moderate, and dense mapped canopy-density classes.

The spatial transition pattern provides further insight into these changes. A considerable proportion of the sanctuary remained within the same mapped canopy-density class between 2016 and 2024, particularly for moderate and dense canopy. The transition analysis identified 795.43 ha of moderate canopy and 1,697.51 ha of dense canopy as persistent classes. At the same time, 308.00 ha transitioned from dense to moderate canopy, while 118.06 ha changed from moderate to dense canopy. A further 146.19 ha transitioned from open to moderate canopy. These contrasting transitions indicate that changes in mapped canopy density were spatially heterogeneous, with both upward and downward class shifts occurring within the sanctuary.The spatial distribution shown in Fig 6 is consistent with this pattern of persistence and localized redistribution. Higher-density canopy remains concentrated in the central and northern portions of the sanctuary, while moderate-density areas occur more extensively across the landscape in 2024. Localized lower-density patches remain visible along peripheral and fragmented areas, indicating that spatial heterogeneity in canopy conditions persists during the post-monsoon period. The observed differences may partly reflect seasonal vegetation phenology and moisture-related changes in spectral response, which can influence the NDVI-based canopy-density classification.

Overall, the post-monsoon comparison does not indicate a simple directional improvement or degradation of canopy condition. Instead, it demonstrates substantial persistence of existing canopy-density patterns accompanied by localized redistribution, particularly between moderate and dense classes. Importantly, these changes represent differences in mapped canopy-density classes and should not be interpreted directly as forest regeneration, degradation, or structural canopy change. The potential influence of differences in Sentinel-2 processing levels between 2016 Level-1C top-of-atmosphere data and 2024 Level-2A surface-reflectance data further warrants cautious interpretation of the interannual differences. Independent field observations and consistently processed surface-reflectance datasets would be required to confirm corresponding biophysical changes.

### 3.5 Canopy density composition and change (2016 vs. 2024)

The comparison of canopy-density composition between 2016 and 2024 showed contrasting interannual patterns between the pre- and post-monsoon seasons (Fig 7). During the pre-monsoon period, the mapped non-forest and open-canopy classes decreased by 39.62% and 7.15%, respectively, while moderate and dense canopy classes increased by 231.64% and 240.43%. In contrast, the post-monsoon period showed a smaller change in non-forest area (+0.67%) and a decline in open canopy (−8.47%), while moderate canopy increased by 26.01% and dense canopy decreased by 9.46%. These results indicate that canopy-density changes were season-dependent and characterized primarily by redistribution among mapped classes rather than a uniform increase in canopy density. The transition analysis further confirmed substantial exchanges between adjacent classes, particularly open-to-moderate and dense-to-moderate transitions. Because the two years were processed using different Sentinel-2 product levels, these interannual differences should be interpreted as changes in mapped canopy-density composition rather than precise estimates of biophysical canopy change.

**Fig 7:**
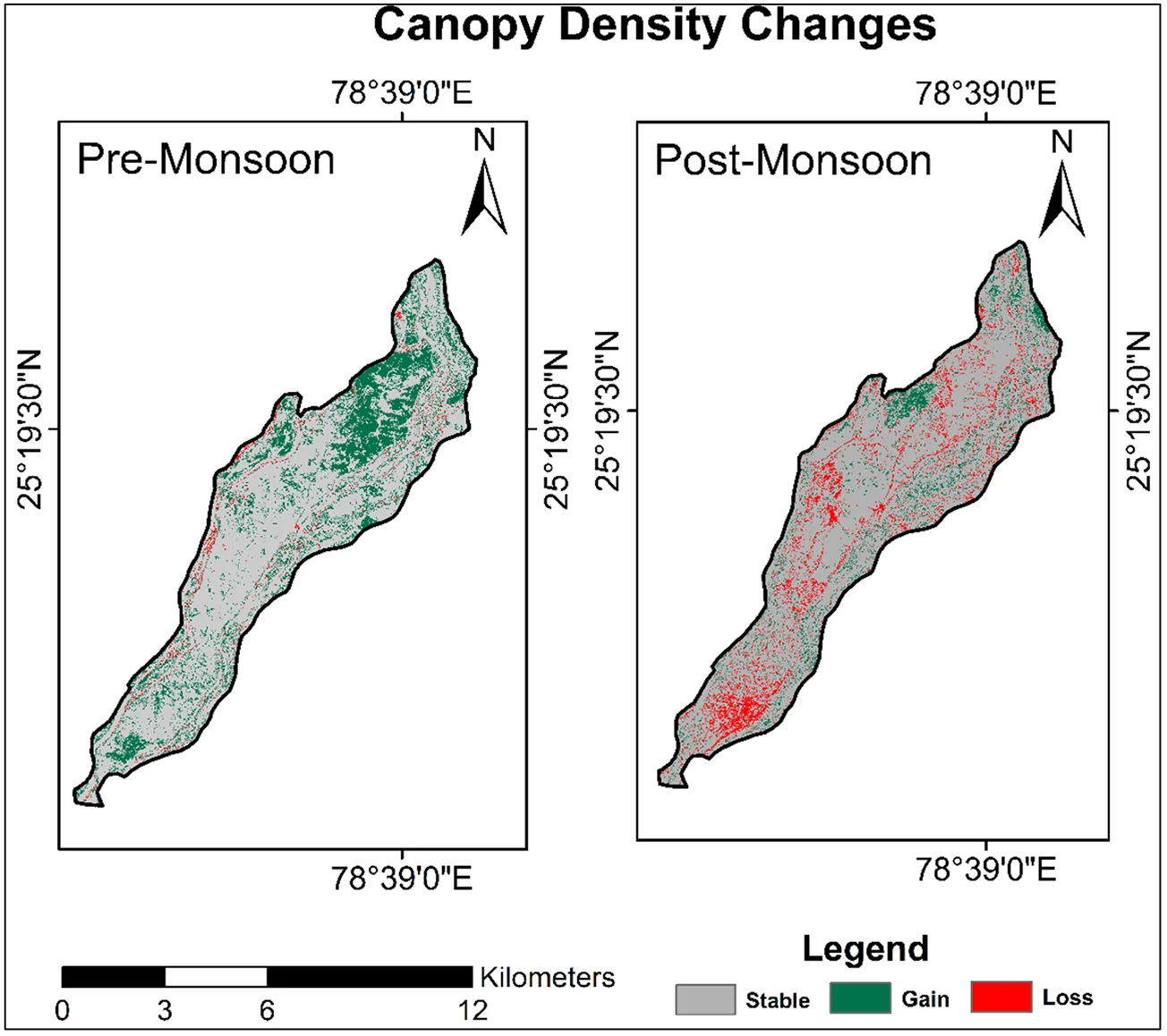
Canopy density changes in different season (Pre-Monsoon and Post-Monsoon seasons).

### 3.6 Area statistics

The spatial extent of the four mapped canopy-density classes was quantified for the 2016 and 2024 classifications during both pre- and post-monsoon periods (Table 4, Fig 8). The total mapped area was 4,533.54 ha for each season-year dataset, allowing direct comparison of the relative distribution of the classified categories. The results indicate substantial seasonal differences in the spatial distribution of canopy-density classes between 2016 and 2024.

**Table 4:** Area and percentage change of mapped canopy-density classes between 2016 and 2024.

Season	Canopy-density class	2016 area (ha)	2024 area (ha)	Change (ha)	Change (%)
**Pre-monsoon**	Non-forest	1,241.92	749.92	−492.00	−39.62
	Open	2,988.16	2,774.40	−213.76	−7.15
	Moderate	271.46	900.27	+628.81	+231.64
	Dense	32.01	108.96	+76.95	+240.43
**Post-monsoon**	Non-forest	676.25	680.77	+4.52	+0.67
	Open	858.05	785.38	−72.67	−8.47
	Moderate	991.73	1,249.70	+257.98	+26.01
	Dense	2,007.52	1,817.69	−189.83	−9.46

**Fig 8:**
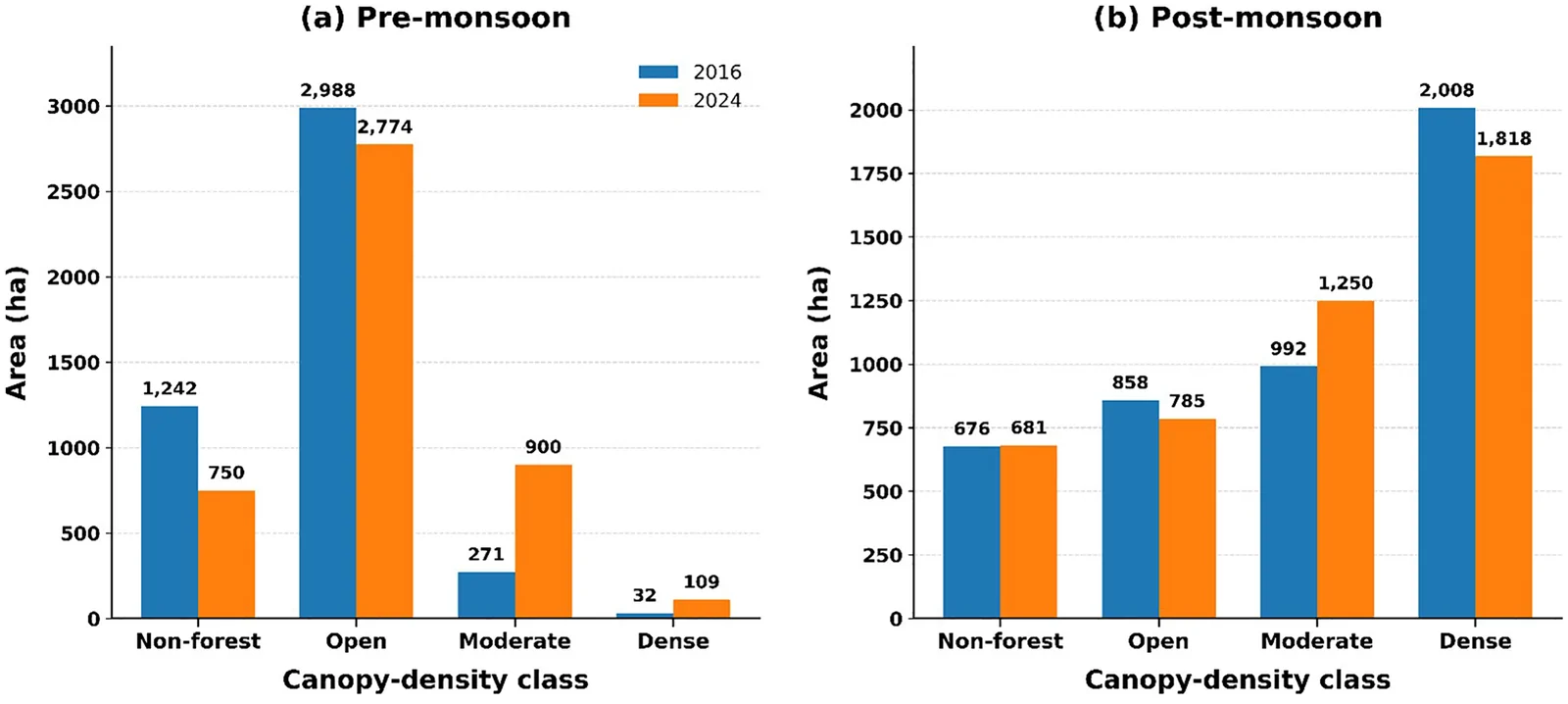
Area distribution of mapped canopy-density classes in 2016 and 2024: (a) pre-monsoon and (b) post-monsoon.

During the pre-monsoon period, the area classified as non-forest decreased from 1,241.92 ha in 2016 to 749.92 ha in 2024, representing a reduction of 39.62%. Open canopy also decreased from 2,988.16 to 2,774.40 ha (7.15% decrease). In contrast, the moderate-canopy class increased substantially from 271.46 to 900.27 ha, corresponding to a 231.64% increase, while the dense-canopy class increased from 32.01 to 108.96 ha (240.43% increase). Thus, the pre-monsoon classification showed a pronounced redistribution of mapped area from non-forest and open-canopy classes toward moderate- and dense-canopy classes.

The post-monsoon results showed a different pattern. The non-forest class remained relatively stable, increasing slightly from 676.25 ha in 2016 to 680.77 ha in 2024 (0.67%). Open canopy decreased from 858.05 to 785.38 ha (8.47% decrease), whereas moderate canopy increased from 991.73 to 1,249.70 ha, representing a 26.01% increase. In contrast to the pre-monsoon results, dense canopy decreased from 2,007.52 to 1,817.69 ha (9.46% decrease). Therefore, the post-monsoon classification was characterized primarily by an increase in the moderate-canopy class accompanied by a modest reduction in dense-canopy extent.

The contrasting seasonal patterns suggest that the mapped canopy-density distribution was strongly influenced by the seasonal spectral response of vegetation. The pre-monsoon results indicate a larger redistribution toward moderate- and dense-canopy classes, whereas the post-monsoon results show greater persistence of the higher-density classes but a shift from dense toward moderate canopy. These differences should therefore be interpreted as **changes in the spatial distribution of mapped canopy-density classes**, rather than direct measurements of changes in structural forest condition.

Because the 2016 classification was based on Sentinel-2 Level-1C top-of-atmosphere reflectance and the 2024 classification used Level-2A surface reflectance, differences in radiometric processing may contribute to the observed interannual variation. Consequently, the reported area changes are best considered **directional changes in mapped classes**, rather than precise estimates of biophysical canopy gain or loss. The transition analysis presented in Section 3.7 further examines the spatial persistence and redistribution underlying these area-level changes.

### 3.7 Canopy-density transition analysis

Canopy-density transitions between 2016 and 2024 were assessed over the common valid comparison area of **4,533.54 ha** for both pre- and post-monsoon periods. The transition analysis quantified the persistence and redistribution of the four mapped canopy-density classes: non-forest, open, moderate, and dense canopy (Table 5; Fig 9). The transition maps were subsequently grouped into five categoriesstable, increase in mapped canopy-density class, decrease in mapped canopy-density class, non-forest to canopy, and canopy to non-forest—to facilitate spatial interpretation.

**Table 5:** Canopy-density transition matrix between 2016 and 2024 for the common valid comparison area (ha).

2016 \ 2024	Non-forest	Open forest	Moderate canopy	Dense canopy
***(a) Pre-monsoon***				
**Non-forest**	698.65	534.61	8.15	0.51
**Open forest**	41.33	2206.16	734.35	6.32
**Moderate canopy**	9.92	33.35	155.42	72.76
**Dense canopy**	0.02	0.27	2.35	29.37
***(b) Post-monsoon***				
**2016 \ 2024**	**Non-forest**	**Open forest**	**Moderate canopy**	**Dense canopy**
**Non-forest**	601.42	74.75	0.08	0.00
**Open forest**	78.65	631.09	146.19	2.12
**Moderate canopy**	0.59	77.64	795.43	118.06
**Dense canopy**	0.12	1.89	308.00	1697.51

**Fig 9:**
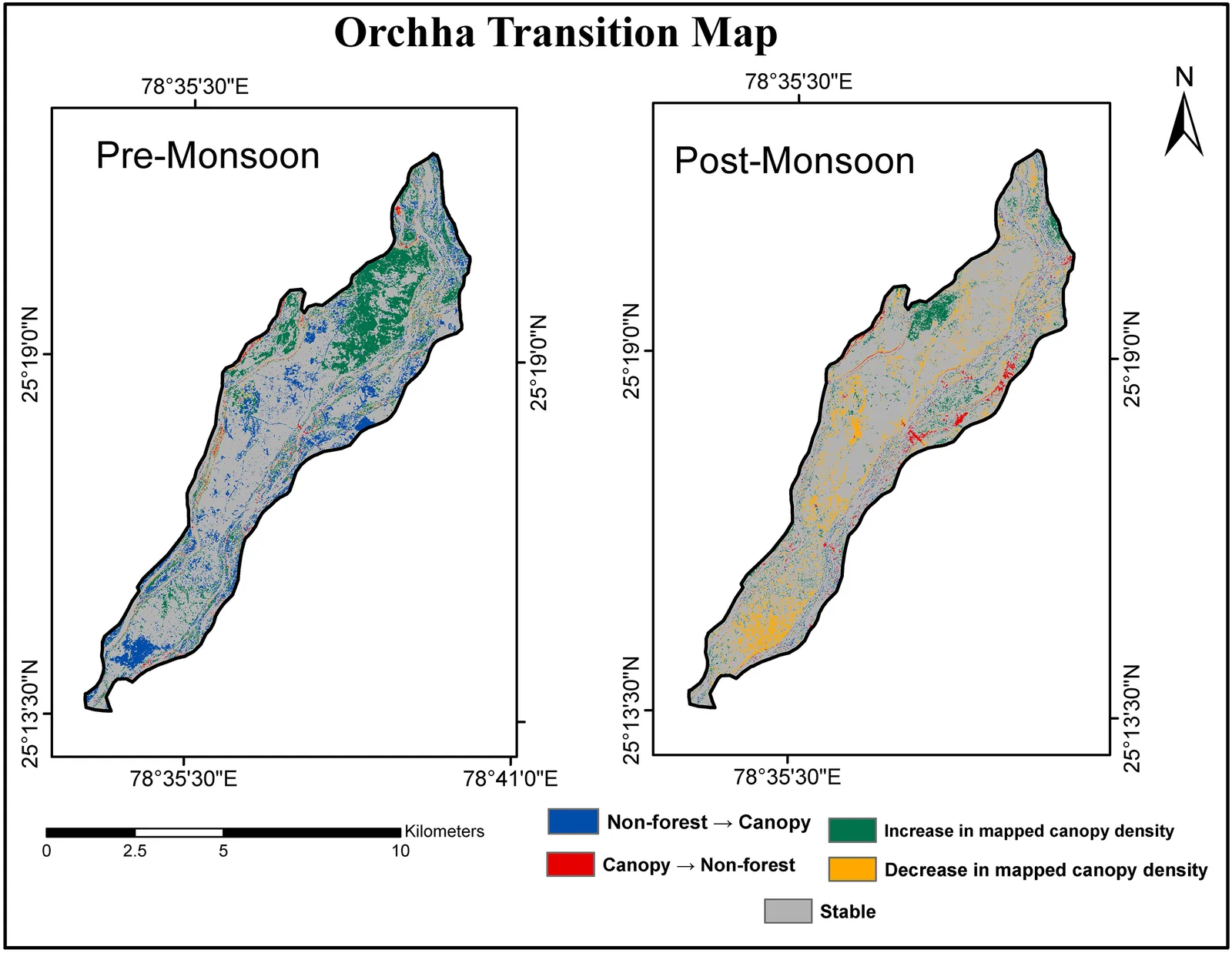
Spatial distribution of canopy-density transitions between 2016 and 2024 during (a) pre-monsoon and (b) post-monsoon periods. Transition categories represent stable classes, increases and decreases in mapped canopy-density class, and transitions between non-forest and canopy classes.

During the pre-monsoon period, substantial redistribution occurred among the lower and intermediate canopy-density classes. The largest transition was from open to moderate canopy (734.35 ha), followed by non-forest to open canopy (534.61 ha). At the same time, 2,206.16 ha of open canopy remained within the open class, indicating considerable class persistence. The moderate class showed 155.42 ha of persistence, while 72.76 ha transitioned from moderate to dense canopy. Transitions directly from non-forest to moderate and dense canopy accounted for 8.15 ha and 0.51 ha, respectively. The dense class retained 29.37 ha of its 2016 extent. Overall, the pre-monsoon pattern was characterized by a pronounced shift from lower-density classes toward moderate and, to a lesser extent, dense mapped canopy classes, together with substantial persistence of the open-canopy class.

The post-monsoon period showed stronger persistence of the higher canopy-density classes. The dense class retained 1,697.51 ha, while 795.43 ha of moderate canopy and 631.09 ha of open canopy remained in their respective classes. Nevertheless, considerable exchange occurred between moderate and dense classes. Approximately 118.06 ha transitioned from moderate to dense canopy, whereas 308.00 ha shifted from dense to moderate canopy. A further 146.19 ha changed from open to moderate canopy. Transitions between non-forest and open canopy were comparatively limited, with 74.75 ha changing from non-forest to open canopy and 78.65 ha from open to non-forest. The relatively large two-way exchange between moderate and dense classes indicates that the post-monsoon spatial pattern was not characterized by a uniform increase in mapped canopy density.

The contrasting transition patterns between the two seasons are also evident in the spatial distribution of the five grouped transition categories (Fig. X). The pre-monsoon map shows more extensive areas associated with transitions toward higher mapped canopy-density classes, particularly within the central and northern portions of the sanctuary. In contrast, the post-monsoon map is dominated by stable areas, with localized patches of both increases and decreases in mapped canopy density. These differences indicate that the spatial expression of canopy-density classes varied between seasons, potentially reflecting seasonal vegetation phenology and moisture conditions.

Importantly, the identified transitions represent changes in mapped canopy-density classes rather than direct evidence of forest regeneration, degradation, or structural canopy change. The 2016 classification was derived from Sentinel-2 Level-1C top-of-atmosphere data, whereas the 2024 classification used Level-2A surface-reflectance data. Consequently, differences in radiometric processing may contribute to the observed interannual class redistribution. The transition results should therefore be interpreted primarily in terms of class persistence and spatial redistribution, while confirmation of corresponding biophysical changes would require consistently processed surface-reflectance data and independent field observations.

### 3.8 Seasonal patterns in canopy dynamics

Seasonal variation strongly influenced the spatial distribution of mapped canopy-density classes in Orchha Wildlife Sanctuary. The pre-monsoon period (March-May) showed a pronounced redistribution toward moderate- and dense-canopy classes between 2016 and 2024, with reductions in non-forest and open-canopy areas. This pattern may reflect differences in vegetation phenology and moisture conditions between the two observation periods; however, the magnitude of the interannual difference may also be influenced by the use of Sentinel-2 Level-1C top-of-atmosphere data in 2016 and Level-2A surface-reflectance data in 2024. Thus, the observed changes should be interpreted as changes in mapped canopy-density classes rather than direct evidence of canopy recovery or structural change.

The post-monsoon period exhibited generally denser mapped canopy conditions than the pre-monsoon period in both years, consistent with seasonal vegetation response following the monsoon. However, the interannual pattern differed from the pre-monsoon comparison: moderate canopy increased by 26.01%, whereas dense canopy decreased by 9.46%. The transition analysis showed that 308.00 ha shifted from dense to moderate canopy, while 118.06 ha transitioned from moderate to dense canopy, indicating substantial two-way redistribution between these classes. Overall, the results demonstrate that canopy-density patterns varied strongly with season and that the 2016–2024 changes were characterized by season-dependent redistribution among mapped canopy-density classes rather than a uniform trajectory of canopy densification.

### 3.9 Spatial distribution of change hotspots

The spatial distribution of canopy-density transitions between 2016 and 2024 showed localized areas of both increase and decrease across Orchha Wildlife Sanctuary (Fig 8). Areas representing transitions toward higher mapped canopy-density classes were distributed mainly across the central and riparian portions of the sanctuary, whereas decreases were more localized along peripheral and fragmented areas. The transition map also showed extensive areas of class persistence, indicating that substantial portions of the sanctuary retained their mapped canopy-density class between the two observation periods.

The spatial pattern varied between seasons. During the pre-monsoon period, transitions toward higher mapped canopy-density classes were more evident in central and riparian areas, consistent with the larger redistribution toward moderate and dense classes observed in the area statistics. During the post-monsoon period, both increases and decreases were spatially distributed, with notable exchanges between moderate and dense canopy classes. These patterns indicate that canopy-density changes were spatially heterogeneous rather than uniformly distributed across the sanctuary.

The concentration of some transitions near riparian areas may be associated with differences in vegetation composition and moisture availability; however, these factors were not independently quantified in the present analysis. Similarly, peripheral transition zones may reflect the heterogeneous nature of vegetation and land-cover conditions along sanctuary boundaries, but specific anthropogenic drivers cannot be established from the classification alone. Therefore, the identified transition hotspots should be regarded as priority areas for field verification and continued monitoring, rather than as confirmed locations of forest regeneration or degradation. Overall, the spatial transition analysis demonstrates the value of seasonal mapping for identifying localized areas of persistent and changing canopy-density classes within the sanctuary.

## 4. Discussion

The spatiotemporal analysis revealed pronounced seasonal and interannual differences in forest canopy-density patterns in Orchha Wildlife Sanctuary. Rather than indicating a uniform increase or decline in canopy condition, the results showed redistribution among the four mapped canopy-density classes. This seasonal dependence is particularly relevant for the dry deciduous forests of the sanctuary, where vegetation greenness and moisture conditions vary substantially with phenological stage. Multi-temporal satellite observations are therefore valuable for distinguishing seasonal vegetation variability from longer-term changes in forest condition [35,38].

During the pre-monsoon period, the strongest interannual redistribution was observed. Non-forest area declined from 1,241.92 ha in 2016 to 749.92 ha in 2024 (−39.62%), while open forest decreased from 2,988.16 to 2,774.40 ha (−7.15%). In contrast, moderate canopy increased from 271.46 to 900.27 ha (+231.64%), and dense forest increased from 32.01 to 108.96 ha (+240.43%). These changes indicate a substantial shift toward higher mapped canopy-density classes. However, the large percentage increases in moderate and dense classes partly reflect their relatively small initial extents in 2016. The transition analysis further showed that the largest pre-monsoon transition was from open forest to moderate canopy (734.35 ha), indicating that much of the observed change occurred through intermediate canopy classes rather than direct conversion from non-forest to dense forest.

The post-monsoon pattern differed considerably. Moderate-canopy area increased from 991.73 to 1,249.70 ha (+26.01%), whereas dense forest declined from 2,007.52 to 1,817.69 ha (−9.46%). Open forest decreased by 8.47%, while non-forest area remained nearly stable (+0.67%). The transition matrix showed that 308.00 ha shifted from dense to moderate canopy, compared with 118.06 ha transitioning from moderate to dense canopy. This redistribution provides a direct explanation for the reduction in total mapped dense-forest area and indicates that the post-monsoon changes involved movement among canopy-density classes rather than simple forest loss.

The contrasting seasonal responses highlight the importance of phenology and moisture conditions when interpreting canopy-density classifications in dry deciduous forests. Sentinel-2 red-edge, near-infrared, and shortwave-infrared observations, together with indices such as NDVI and NDMI, can capture seasonal variation in vegetation greenness and moisture status [37,38]. The lower pre-monsoon canopy density observed in 2016 is consistent with possible antecedent moisture stress, while the higher mapped moderate- and dense-canopy extent in 2024 may reflect differences in moisture availability, vegetation condition, and disturbance pressure. However, rainfall and drought effects were not quantitatively modelled in this study; therefore, these factors should be regarded as possible explanations rather than demonstrated causal drivers.

Spatially, transitions toward higher mapped canopy-density classes were concentrated mainly in central and riparian portions of the sanctuary, whereas localized declines occurred more frequently near peripheral areas, settlements, agricultural boundaries, and access routes. The concentration of higher canopy classes along the Betwa River corridor is consistent with the ecological importance of riparian environments and their greater moisture availability. Nevertheless, the present analysis did not independently quantify hydrological conditions or anthropogenic disturbance, and these interpretations therefore require field verification.

The Random Forest models produced high agreement with the adopted NDVI-informed canopy strata, with overall accuracies ranging from 97.80% to 98.95% and Kappa coefficients from 0.970 to 0.986. These values indicate strong classification consistency; however, because NDVI was used to define the canopy-density strata and was also included as a predictor, the accuracy values should not be interpreted as independent validation of field-measured canopy structure.

An additional limitation is the difference in Sentinel-2 processing levels. The 2016 composites were derived from Level-1C top-of-atmosphere reflectance, whereas the 2024 composites used Level-2A surface reflectance. Although cloud masking, seasonal median compositing, and normalized vegetation indices reduced some sources of variability, differences in atmospheric correction may still influence canopy-class assignment. Therefore, the interannual differences should be interpreted as changes in mapped canopy-density classes rather than precise quantitative changes in biophysical canopy structure. Despite this limitation, the GEE-based framework provides a reproducible approach for identifying spatially dynamic canopy areas and supporting forest monitoring and conservation planning.

### 4.1 Limitations

Several limitations of the present analysis should be recognised. The canopy density classes were delimited from NDVI, and NDVI-based stratification carries well-known constraints. NDVI saturates at high leaf area index, so it discriminates poorly among the densest stands and can compress real structural differences within the high canopy density class. It is also sensitive to soil background reflectance and to understorey greenness wherever crown closure is incomplete, which introduces uncertainty at the boundary between the non-forest and low canopy density classes, particularly over the shallow, light-toned Vindhyan soils of the northern blocks. Because the constituent species are strongly deciduous, NDVI in this landscape is a function of phenological state as well as of structure, so a given NDVI value does not correspond to a fixed crown density across seasons; this is the reason the pre-monsoon and post-monsoon composites were classified with separate models rather than pooled. Finally, NDVI records horizontal greenness rather than vertical structure, so the density classes reported here should be read as strata of canopy cover and not as measures of stand height, basal area or biomass.The common valid comparison area used for transition analysis was 4,533.54 ha for both seasonal comparisons; therefore, transition matrices represent only pixels with valid classifications in both years and should not be interpreted as independent estimates of total sanctuary area.

Second, validation was carried out against reference points labelled by visual interpretation of high-resolution imagery rather than against field measurements. No ground photographs, GPS-located field plots or field-measured crown density values were collected during this study, and the accuracy figures reported in Section 3.1 therefore express agreement with photo-interpreted reference data rather than with independently measured canopy conditions. Photo-interpretation is reliable for separating the extremes of the density gradient, but it is least reliable precisely where the classifier is weakest, at the boundary between the low and moderate classes, so the accuracies reported for those classes should be treated as upper estimates. A field campaign establishing permanent plots with GPS-located canopy density measurements, representative ground photographs for each density class and stand-level records of dominant species is required to convert these results from a photo-validated to a field-validated product, and is planned as the next stage of this work.

Third, the two dates were not acquired at the same processing level. The 2024 composites were built from Level-2A surface reflectance products, whereas Level-2A products were not available in the archive for 2016, so the 2016 composites were built from Level-1C top-of-atmosphere reflectance. Top-of-atmosphere reflectance retains both the additive effect of atmospheric path radiance and the multiplicative effect of atmospheric transmittance. Both are wavelength dependent; both are stronger in the red than in the near-infrared, and both therefore act to depress NDVI relative to a surface reflectance retrieval; the resulting bias is systematic rather than random, and differences in atmospheric correction may alter the distribution of NDVI and NDMI values between the two processing levels and can therefore influence the assignment of pixels to canopy-density classes. The direction and magnitude of this effect cannot be quantified from the present analysis. Consequently, the 2016–2024 area differences should be interpreted as changes in mapped classes rather than as exact estimates of biophysical canopy change. Three measures were taken to limit this effect. Cloud and cirrus contaminated pixels were removed with the QA60 mask before compositing, so that the residual atmospheric signal is that of clear-sky conditions rather than of haze and thin cloud. Seasonal median compositing was applied, which suppresses date-specific atmospheric outliers by taking the central value of the clear-sky stack rather than relying on a single acquisition. The classification was driven by normalised ratio indices, NDVI and NDMI, rather than by raw band values, and ratio formulations partially cancel the multiplicative component of the atmospheric effect, although they do not cancel the additive path radiance component. These measures reduce but do not eliminate the inconsistency, and the residual bias remains a genuine limitation of the 2016–2024 comparison. Its direction should be borne in mind when the magnitude of change is interpreted: because an uncorrected 2016 scene should appear less densely vegetated than it actually is, direction and magnitude of any residual radiometric bias cannot be quantified from the present analysis and should therefore be considered when interpreting the interannual differences.

## 5. Future work

Future studies should incorporate field-measured canopy cover, repeated ground observations, and additional multi-temporal Sentinel-2 observations to strengthen calibration and validation of the canopy-density classes. Harmonized surface-reflectance products across all observation years would also improve the comparability of long-term change estimates. Integration of LiDAR or other three-dimensional observations could further distinguish canopy structure from spectral greenness and improve assessment of vertical forest structure. Multi-year time series and climate datasets could additionally be used to quantify the relationships among rainfall variability, drought, phenology, and canopy dynamics.

## 6. Conclusion

This study evaluated seasonal and interannual forest canopy-density dynamics in Orchha Wildlife Sanctuary using Sentinel-2 imagery, vegetation indices, GLCM-based texture information, topographic variables, and Random Forest classification implemented in Google Earth Engine. The results demonstrated clear seasonal and interannual redistribution among the four mapped canopy-density classes. During the pre-monsoon period, non-forest and open-canopy areas decreased by 39.62% and 7.15%, respectively, while moderate and dense mapped canopy classes increased by 231.64% and 240.43%. During the post-monsoon period, moderate canopy increased by 26.01%, whereas dense canopy decreased by 9.46%, indicating that changes were not uniformly directional across seasons. Within the common valid comparison area of 4,533.54 ha, transition analysis identified substantial class persistence and redistribution, including 734.35 ha of open-to-moderate transition during the pre-monsoon period and 308.00 ha of dense-to-moderate transition during the post-monsoon period. These findings demonstrate the importance of seasonal observations for characterizing canopy-density patterns in seasonally dry deciduous forests. However, because 2016 and 2024 were analyzed using Sentinel-2 Level-1C top-of-atmosphere and Level-2A surface-reflectance products, respectively, the interannual differences should be interpreted as changes in mapped canopy-density classes rather than precise biophysical changes in canopy structure. The GEE-based workflow provides a reproducible approach for seasonal canopy monitoring and for identifying spatially dynamic areas that may support conservation monitoring and management prioritization in protected landscapes.

The proposed STGCNet framework also provides scope for future integration with UAV-based high-resolution mapping and autonomous remote sensing systems. Its capability to extract spatially detailed land-cover information could support UAV-assisted environmental monitoring, particularly in heterogeneous and dynamically changing landscapes. Recent advances in resilient UAV localization further demonstrate the potential for combining high-resolution visual perception with autonomous positioning and navigation capabilities [47]. Such integration could enable intelligent, flexible, and near-real-time geospatial monitoring for applications in forest management, ecosystem assessment, and precision land-resource management.
